# Mapping epigenetic modifications by sequencing technologies

**DOI:** 10.1038/s41418-023-01213-1

**Published:** 2023-09-01

**Authors:** Xiufei Chen, Haiqi Xu, Xiao Shu, Chun-Xiao Song

**Affiliations:** 1 Ludwig Institute for Cancer Research, Nuffield Department of Medicine, University of Oxford, Oxford, OX3 7FZ UK; 2 Target Discovery Institute, Nuffield Department of Medicine, University of Oxford, Oxford, OX3 7FZ UK

**Keywords:** Epigenetics, Chemical genetics

## Abstract

The “epigenetics” concept was first described in 1942. Thus far, chemical modifications on histones, DNA, and RNA have emerged as three important building blocks of epigenetic modifications. Many epigenetic modifications have been intensively studied and found to be involved in most essential biological processes as well as human diseases, including cancer. Precisely and quantitatively mapping over 100 [1], 17 [2], and 160 [3] different known types of epigenetic modifications in histone, DNA, and RNA is the key to understanding the role of epigenetic modifications in gene regulation in diverse biological processes. With the rapid development of sequencing technologies, scientists are able to detect specific epigenetic modifications with various quantitative, high-resolution, whole-genome/transcriptome approaches. Here, we summarize recent advances in epigenetic modification sequencing technologies, focusing on major histone, DNA, and RNA modifications in mammalian cells.

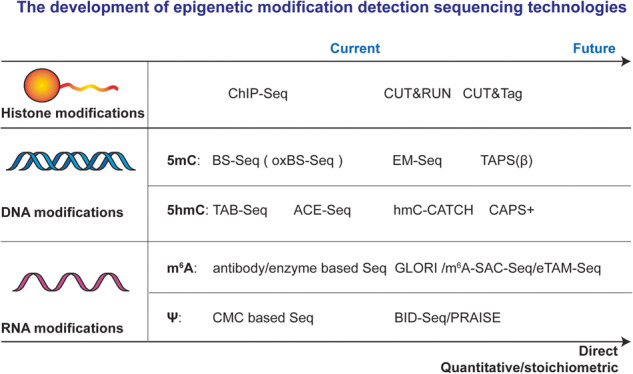

## Facts

- Traditional, bisulfite sequencing is the gold standard in DNA methylation sequencing but it severely damages DNA [4].
- Recently, EM-Seq and TAPS have been developed to replace bisulfite sequencing [5, 6].
- Very recently, base-resolution and quantitative RNA epitranscriptomic modifications sequencing methods have started to emerge [7–11].
- Third-generation sequencing is promising for epigenetic sequencing; however, many challenges remain to be resolved [12].

## Open questions

- Could further RNA modification sequencing methods be developed with improved efficiency and accuracy?
- Could simultaneous base-resolution sequencing of a wide range of epigenetic modifications be achieved?
- Could third-generation sequencing finally deliver epigenetic data as accurate and cost-effective as next-generation sequencing?
- Could we develop large-scale live cell temporal/spatial epigenetic sequencing?

## Introduction

**Fig. 1:**
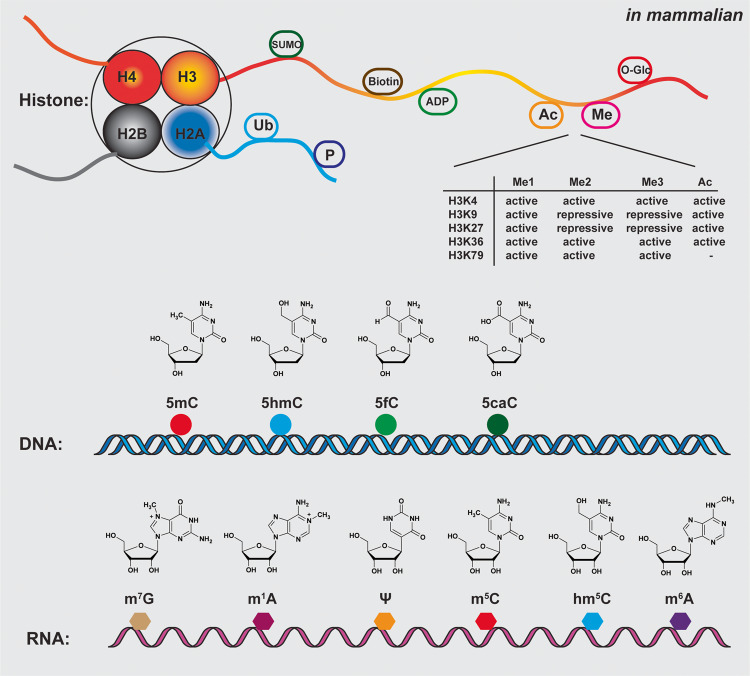
Epigenetic modifications in Histone, DNA and RNA. Histone Modifications: Nucleosomes are composed of DNA wrapped around the four core histones (H3, H4, H2A, H2B). Post-translational modifications, including acetylation (Ac), methylation (Me), phosphorylation (P), ubiquitylation (Ub), SUMOylation (SUMO), ADP ribosylation (ADP), O-GlcNAcylation (O-Glc), and biotinylation (Biotin), are commonly observed on the N-terminal histone tails. Notably, significant modifications of Histone H3 have been associated with either active or repressive gene expression. Major DNA Modifications in Mammals: DNA modifications include DNA 5-methylcytosine (5mC), 5-hydroxymethylcytosine (5hmC), 5-formylcytosine (5fC), and 5-carboxylcytosine (5caC). These modifications possess distinct chemical structures and are crucial in epigenetic regulation. Major RNA modifications in Mammals: RNA modifications include *N*^6^-methyladenosine (m^6^A), pseudouridine (Ψ), *N*^1^-methyladenosine (m^1^A), *N*^7^-methylguanosine (m^7^G), 5-methylcytidine (m^5^C) and 5-hydroxymethylcytidine (hm^5^C) modifications, and their chemical structures.

In 1942, embryologist Conrad Waddington first established the concept of “epigenetics” with the famous “epigenetic landscape” model [13]. However, the explosion of epigenetic studies has only occurred over the last two decades. The term “epigenetic” refers to the alteration of gene expression without change of the DNA sequence, which mainly occurs in the form of a myriad of chemical modifications in histone, DNA, and RNA. Among them, chromatin structure modification studies started in the 1990s. Over 100 distinct modifications have been found in histone, including acetylation (Ac), methylation (Me), phosphorylation (P), ubiquitylation (Ub), SUMOylation (SUMO), ADP ribosylation (ADP), O-GlcNAcylation (O-Glc), and biotinylation (Biotin) (Fig. 1) [1, 14, 15]. In the late 1940s, 5-methylcytosine (5mC) was the first identified DNA chemical modification [16], and to date, over 17 types of DNA chemical modifications have been identified [2]. 5mC is the most predominant and important modification in mammalian DNA, the so-called “fifth base”. Its oxidative products, 5-hydroxymethylcytosine (5hmC), 5-formylcytosine (5fC), and 5-carboxylcytosine (5caC), also exist in mammalian DNA (Fig. 1). Compared to DNA, RNA modifications are more diverse, with over 160 types of RNA modifications reported thus far [3]. The most common dynamic RNA modifications include *N*^6^-methyladenosine (m^6^A), pseudouridine (Ψ), *N*^1^-methyladenosine (m^1^A), *N*^7^-methylguanosine (m^7^G), 5-methylcytidine (m^5^C) / 5-hydroxymethylcytidine (hm^5^C) in mammalian cells, leading to the exciting field of epitranscriptomics [17–20] (Fig. 1). In the past two decades, the dysregulation of epigenetic modifications has been revealed in many studies and serves as a hallmark of cancer: many somatic mutations in human cancers occur in epigenetic regulators [21–23].

The first wave of methods to identify these epigenetic modifications used bulk measurements such as thin layer chromatography (TLC), LC/GC-MS, immunofluorescence, and immunoprecipitation. These methods, however, could not provide sequence information about the modifications [24]. With the rapid development of next generation sequencing (NGS), sequencing based detection methods for epigenetic modifications have been developed at a rapid pace. Earlier methods usually relied on affinity enrichment (i.e. immunoprecipitation, biotin pull-down, etc.). While useful and cost-effective, they only provide limited semi-quantitative and low-resolution (a few hundred base pairs) information about the modification. More recent developments focus on high-resolution (e.g., base-level resolution) and quantitative sequencing methods, which provide a more complete picture of the modification. While NGS has significantly advanced the field, it still has limitations, including short read lengths, biases introduced by amplification steps, and difficulties in accurately resolving repetitive genomic regions. In this regard, the future development of third-generation sequencing emerges as a promising solution [25, 26]. In this review, we summarize the diversity and complexity of epigenetic modifications and their related sequencing methods, focusing on recent advances in quantitative and base-resolution technologies for major DNA (5mC/5hmC/5fC/5caC) and RNA (m^6^A/Ψ) modifications in mammalian cells. This review is an update and extension of our previous summary on DNA and RNA modification detection [19].

## Histone modification detection by sequencing technologies

**Fig. 2:**
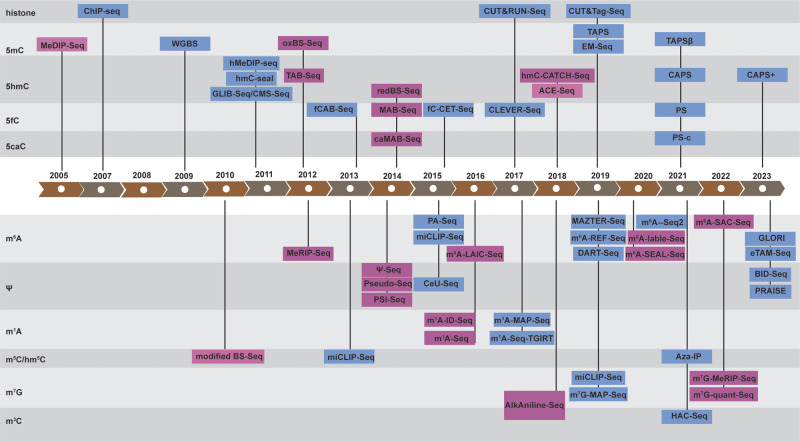
Timeline landscape of epigenetic modification detection technologies based on next-generation sequencing. A comprehensive overview of the progression of next-generation sequencing techniques developed over time for detecting major Histone, DNA (5mC, 5hmC, 5fC, 5caC) and RNA (m^6^A, Ψ, m^1^A, m^5^C/ hm^5^C, m^7^G, m^3^C) modifications.

Chromatin is composed of DNA and histone proteins with nucleosomes as the basic structural units. Around 147 base pairs of DNA are packaged into an octamer of the four core histones (H3, H4, H2A, H2B) as the nucleosome [27]. Histone modifications usually occur at the unstructured N-terminal of the histones, which could modify chromatin accessibility to chromatin remodeling enzymes and transcription factors, thus regulating gene expression [14, 28]. Some modifications such as H3K27ac and H3K4me1/2/3 are active markers, while others such as H3K27me3 and H3K9me3 are repressive markers in histone H3 tail (Fig. 1) [14, 29–32]. It has been widely established that dysregulation of histone modifications causes a number of diseases, including cancer [30, 33, 34]. It is therefore important to study their location and distribution across the genome. Among many approaches to mapping these modifications, chromatin immunoprecipitation followed by sequencing (ChIP–Seq) is the most classical method to sequence histone modifications in a genome-wide manner. ChIP–Seq is based on formaldehyde/paraformaldehyde-mediated protein-DNA crosslinking, followed by incubation with the specific antibodies to enrich the target histone modification and finally library construction for next-generation sequencing to detect genome-wide histone modification distribution. The earliest formaldehyde-mediated protein-DNA crosslinking ChIP experiment was conducted by Solomon et al. [35] to probe histone H4 and hsp70 DNA interactions in vivo in 1988, whilst the first ChIP-Seq method was established by Barski et al. in 2007, who mapped genome-wide distributions of 20 histone lysine and arginine methylations [36]. Despite this technique being widely used, ChIP–Seq has several limitations, such as the requirement for larger amounts of input DNA, false positive rates induced by cross-linking between DNA and protein, and high background noise due to poor antibody specificity. [37]. Recently, two novel technologies have been established to overcome these limitations. In 2017, Cleavage Under Targets and Release Using Nuclease (CUT&RUN) technology was described, for semi-quantitative detection of protein-DNA interactions in situ at ~20 bp resolution [38, 39] (Fig. 2). To avoid the epitope masking and false positive binding sites generated by the crosslinking step in ChIP-Seq, CUT&RUN immobilizes the cells on lectin-coated magnetic beads and incubates with specific antibodies and protein A-MNase. Ca^2+^ was then added to initiate the cleavage reaction to release the target protein-DNA complexes for sequencing. In 2019, an improved Cleavage Under Targets and Tagmentation (CUT&Tag) technology was developed, which replaces the MNase digestion with Tn5 tagmentation (Fig. 2). CUT&Tag simplifies the library construction steps [40], making it more feasible for single-cell experiments to probe histone modifications, such as those that are characteristic of active promoters (H3K4me3), enhancers (H3K27ac), gene bodies (H3K36me3) and inactive regions (H3K27me3), as demonstrated in mice brains [41]. However, CUT&RUN and CUT&Tag inherit the disadvantages of antibody-based approaches. Future development could focus on novel antibody-free and enrichment-free approaches to map histone modifications quantitatively at single base resolution.

## DNA methylation (5mC) sequencing technologies

DNA methylation of cytosine (5mC) is the most predominant modification, and accounts for ~5% of all cytosine (C) [42]. 5mC modification on DNA is catalyzed by a family of enzymes known as DNA methyl transferases (DNMTs), predominantly in the symmetrical CpG dinucleotides: 70–80% of CpGs in the mammalian genome are methylated [43]. DNA methylation has been found to relate to X chromosome inactivation, and genomic imprinting, and is dysregulated in most diseases, including cancer [33, 34, 44]. The hypermethylation of tumor suppressor genes or hypomethylation of tumor oncogenes has been commonly identified in many cancers [45–47]. This makes DNA methylation an attractive therapeutic target for cancer treatment. For example, DNA methylation inhibitors, such as 5-azacytidine, 5-aza-2’-deoxycytidine (decitabine, DAC), and cytarabine (Ara-C), have been widely used to inhibit tumor growth in vivo and also applied to treat myelodysplastic syndromes (MDS) and acute myeloid leukemia (AML) patients [48–52].

**Fig. 3:**
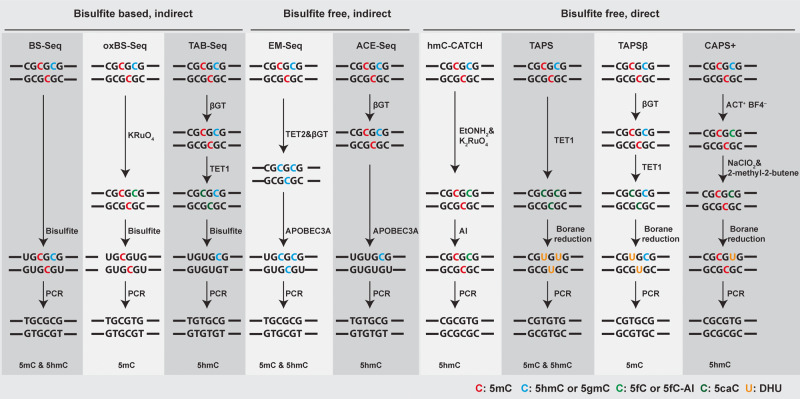
DNA 5mC and 5hmC modification detection sequencing technologies. Summary of prominent techniques employed in the detection of DNA 5mC and 5hmC epigenetic modifications. Bisulfite-based and indirect methodologies encompass bisulfite sequencing (BS-Seq) for both 5mC and 5hmC, oxidative bisulfite sequencing (oxBS-Seq) for 5mC, and TET-assisted bisulfite sequencing (TAB-Seq) for 5hmC. Bisulfite-free yet indirect methods include enzymatic methyl-Seq (EM-Seq) for both 5mC and 5hmC and APOBEC-coupled epigenetic sequencing (ACE-Seq) for 5hmC. Alternatively, bisulfite-free and direct strategies entail chemical-assisted C-to-T conversion of 5hmC sequencing (hmC-CATCH) for 5hmC, TET-assisted pyridine borane sequencing (TAPS) for both 5mC and 5hmC, TAPSβ for 5mC, chemical-assisted pyridine borane sequencing plus (CAPS+) for 5hmC.

In 2005, MeDIP-Seq (Fig. 2) was developed to capture differential DNA methylation in normal and transformed human cells [53]. However, as it is based on antibody enrichment, it suffers from the same limitations as ChIP-Seq. In 1992, bisulfite treatment for 5mC detection was first developed by Frommer, et al. [54]. This method utilizes sodium bisulfite, which specifically deaminates unmodified C to Uracil (U) while leaving 5mC and 5hmC intact. During the subsequent PCR and sequencing, U is therefore read as thymine (T). When comparing bisulfite-converted sequences with the reference genome, 5mC and 5hmC can be distinguished from unmodified C. Based on this bisulfite reaction, the base-resolution and quantitative whole-genome bisulfite sequencing (WGBS) (Fig. 2) for 5mC and 5hmC mapping was developed in 2009 [55]. With over 99% conversion of unmodified C to U, WGBS became the powerful and widely used gold standard for mapping 5mC and 5hmC (Fig. 3). However, WGBS has two significant drawbacks. First, bisulfite treatment is a very harsh chemical reaction that will cause severe DNA damage and loss [4]. Second, the converted unmodified cytosine, which accounts for around 95% of all cytosine in the genome, leads to reduced DNA sequence complexity, lower mapping efficiency, and biased genomic coverage after bisulfite treatment. Nevertheless, various improvements have been made, such as post-bisulfite adaptor tagging (PBAT), which salvages fragmented DNA caused by bisulfite treatment to mitigate the bisulfite-induced loss of intact sequencing templates [56]. By adopting PBAT, various single-cell WGBS protocols have been developed [57, 58].

To overcome the limitations of WGBS, two bisulfite-free DNA methylation sequencing methods have recently been developed. The enzymatic methyl-Seq (EM-Seq) (Fig. 2) is an enzymatic deamination method involving three enzymes in two reactions [6]. In the first reaction, Tet methylcytosine dioxygenase 2 (TET2) is used to catalyze the oxidization of 5mC to 5hmC, 5fC, and 5caC. In the same reaction, β-glucosyltransferase (βGT) glucosylates both TET2-derived and genomic 5hmC to form 5-(β-glucosyloxymethyl) cytosine (5gmC). In the second reaction, AID/APOBEC family DNA deaminase APOBEC3A deaminates unmodified C to U, while 5fC, 5caC, and 5gmC are protected from APOBEC3A deamination (Fig. 3). EM-Seq achieved over 96% protection rate on 5mC with less than 0.6% non-conversion rate (false positive rate) of unmodified cytosine. The mild enzymatic reactions make EM-Seq compatible with input DNA quantities as low as 100 pg. Recently, single-cell EM-Seq has also been established [59]. Similar to WGBS, EM-Seq indirectly maps the 5mC and 5hmC modifications by converting unmodified cytosine in DNA, which leads to a low complexity genome.

Unlike WGBS and EM-Seq, TET-assisted pyridine borane sequencing (TAPS) (Fig. 2) developed by Liu et al. [5] in 2019 is a direct DNA 5mC and 5hmC detection method. It is based on a novel borane reduction chemistry to convert 5caC to dihydrouracil (DHU), which is a mild reaction with little DNA damage compared to the bisulfite reaction. TAPS combines ten-eleven translocation (TET) oxidation of 5mC and 5hmC to 5caC and borane reduction conversion of 5caC to DHU, which subsequently reads as T after PCR amplification (Fig. 3). TAPS achieved a high conversion rate of over 96% on 5mC and a low false positive rate of 0.23% on unmodified cytosine. A key advantage of TAPS is that it induces a C-to-T transition only at modified cytosine (5mC and 5hmC), which only accounts for 5% of all cytosine. Such direct detection preserves the underlying genomic information, enabling TAPS to achieve substantially higher mapping rate and sequencing quality at half the sequencing cost to WGBS [60]. By incorporating βGT to convert 5hmC to 5gmC and to protect 5hmC from TET oxidation and borane reduction, Liu et al. extended TAPS to TAPSβ to enable 5mC-specific sequencing (Fig. 3) [61].

## DNA methylation oxidative products: 5hmC, 5fC, and 5caC sequencing technologies

The removal of 5mC in cells is accomplished by TET family proteins [62, 63] or passively by DNA replication [64, 65]. While TET1/2/3 show tissue specific distributions, they can all convert 5mC to 5hmC, 5fC, and 5caC in three consecutive steps. 5fC and 5caC can be excised and restored to C through thymine DNA glycosylase (TDG) and base excision repair (BER) pathway, resulting in active demethylation [66]. 5hmC was first identified in 2009 [62, 67] and is highly abundant in neurons and related to active genes, such as *Pcp4*, *Neurod2* [68]. 5fC and 5caC have much lower abundances in the mammalian genome and are considered to be demethylation intermediates, however, recent investigations also showed the possibility of these modifications being stable in nature [69, 70]. 5hmC plays an important role in many biological processes such as zygote/embryonic development [71, 72], and cell differentiation [73], and its dysregulation has been shown in tumorigenesis [74, 75], while the 5fC and 5caC’s role in cells remain to be delineated.

To map genome-wide 5hmC distribution, various sequencing methods have been developed, such as hMeDIP-Seq [76], hmC-seal [77], CMS-Seq and GLIB-Seq (Fig. 2), all of which are antibody or biotin based affinity enrichment of 5hmC-containing genomic DNA with limited resolution. To map 5hmC quantitatively across the whole genome at the single base resolution, several technologies have been developed, including oxBS-Seq, TAB-Seq [78–81] in 2012, ACE-Seq [82], hmC-CATCH-Seq [83] in 2018, CAPS [61] in 2021, and CAPS+ [84] in 2023 (Fig. 2). Oxidative bisulfite sequencing (oxBS-Seq) utilizes potassium perruthenate (KRuO_4_) oxidation of 5hmC to 5fC to remove the 5hmC signal from WGBS, thereby only detecting 5mC (Fig. 3). By comparing and subtracting the result of oxBS from WGBS, the base-resolution 5hmC level can be obtained. oxBS-Seq was employed in mouse embryonic stem (ES) cells, leading to the identification of approximately 800 5hmC-modified CpG islands (CGIs), exhibiting an average hydroxymethylation level of 3.3%. Notably, the highly modified CGIs discovered through this method were correlated to intragenic and intergenic CGIs, but not transcription start site (TSS) CGIs [80]. TET-assisted bisulfite sequencing (TAB-Seq), on the other hand, uses TET oxidation and βGT glucosylation to eliminate the 5mC signal from WGBS, thereby detecting 5hmC directly without the need to compare with WGBS (Fig. 3). Utilizing TAB-Seq on mouse embryonic stem cells (mES), a total of 2 057 636 5hmC sites were identified. These sites are found in close proximity to, but not directly on, transcription factor-binding sites. Moreover, the distribution of these sites demonstrates significant variation across different distal-regulatory elements [79]. Both oxBS-Seq and TAB-Seq are based on bisulfite sequencing and have therefore inherited the disadvantages of WGBS, namely severe loss of DNA integrity and complexity.

Recently, four bisulfite-free base-resolution 5hmC sequencing methods have been developed. In 2018, Chemical-assisted C-to-T conversion of 5hmC sequencing (hmC-CATCH) was developed [83], using potassium ruthenate (K_2_RuO_4_) oxidation of 5hmC to 5fC, followed by chemical labeling of 5fC by an azido derivative of 1,3-indandione (AI). The labeled adduct is read as T after PCR amplification, making hmC-CATCH a direct sequencing method of 5hmC (Fig. 3). However, hmC-CATCH is not quantitative due to the use of a biotin pull-down enrichment step. Utilizing the hmC-CATCH technique, 607 021 5hmC sites were detected in human embryonic stem cells, and it is also the first time to unveil the base-resolution hydroxymethylome in the cell-free DNA (cfDNA) of both healthy individuals and individuals diagnosed with cancer [83]. The second of these methods, APOBEC-coupled epigenetic sequencing (ACE-Seq), is an enzymatic deamination method for sequencing 5hmC like EM-Seq. It uses βGT to block 5hmC as 5gmC, before APOBEC3A deamination of unmodified C and 5mC to U. ACE-Seq is nondestructive and can achieve a 98.5% protection rate on 5hmC with 0.1% and 0.5% non-conversion rates (false positive rate) on unmodified cytosine and 5mC, respectively (Fig. 3). ACE-Seq identified 798 643 5hmC sites in mES and also provided valuable insights into the distribution of 5hmC in cortical excitatory neurons [82]. Thirdly, in 2021, chemical-assisted pyridine borane sequencing (CAPS) was developed as a sister method for TAPS. CAPS utilizes potassium ruthenate oxidation of 5hmC to 5fC, followed by borane reduction of 5fC to DHU (Fig. 3). It achieved 83.1% 5hmC-to-T conversion, 0.72% and 0.38% false-positive rates on unmodified cytosine and 5mC, respectively. Owing to the direct 5hmC readout, CAPS showed improved sequence complexity, higher base quality, and mapping rate compared to TAB-Seq and ACE-Seq. CAPS detected 1 762 287 5hmC sites in mES [61]. Finally, in 2023, chemical-assisted pyridine borane sequencing plus (CAPS+) [84] was developed as an updated version of CAPS. CAPS+ replaced potassium ruthenate oxidation in CAPS with two milder chemical oxidation reactions: using 4-acetamido-2,2,6,6-tetramethylpiperidine-1-oxoammonium tetra-fluoroborate (ACT^+^ BF4^−^) to oxidize 5hmC to 5fC, then employing sodium chlorite (NaClO_2_) in the Pinnick oxidation to convert 5fC into 5caC. Borane reduction converts 5caC to DHU, which is similar to the process used in CAPS (Fig. 3). The CAPS+ builds upon the strengths of CAPS, with enhancements in conversion rate (achieving 94.5% for 5hmC) and reductions in false-positive rates (0.15% for 5mC and 0.17% for unmodified cytosine) [84].

Beyond 5hmC, various 5fC and 5caC sequencing methods have also been developed [85]. Several bisulfite-based 5fC and 5caC sequencing methods have been developed utilizing various approaches to modulate the behavior of 5fC and 5caC in bisulfite mediated deamination. This includes 5fC chemically assisted bisulfite sequencing method (fCAB-Seq) [86], reduced bisulfite sequencing (redBS-Seq) [87], M.SssI methylase-assisted bisulfite sequencing (MAB-Seq) and caMAB-Seq [88, 89] (Fig. 2). The investigations presented in these studies uncovered the preferential occurrence of 5fC at poised enhancers, highlighting the crucial involvement of TDG in active DNA demethylation process. Additionally, these studies also detected a notable asymmetry between the strands for both 5fC and 5caC in mES [87, 88]. In 2015, the bisulfite-free cyclization-enabled C-to-T transition of 5fC sequencing (fC-CET-Seq) [90] (Fig. 2) was developed utilizing an azido derivative of 1, 3-indandione to convert 5fC to an adduct that can be read as T following PCR amplification. In 2017, it was further developed into chemical-labeling-enabled C-to-T Conversion Sequencing (CLEVER-Seq) (Fig. 2) for single-cell 5fC sequencing [91]. CLEVER-Seq unraveled the inherent heterogeneity of 5fC in mES. Additionally, 5fC exhibited parental-specific patterns, and its localization on promoters correlated with gene activation throughout the preimplantation development of mice. Based on the borane reduction chemistry, Liu et al. also developed pyridine borane sequencing (PS) and pyridine borane sequencing for carboxylcytosine (PS-c) (Fig. 2) for whole-genome base-resolution sequencing of 5fC and 5caC [61].

## Other DNA modifications (6mA, 4mC, base J)

Beyond 5mC, two other DNA methylation forms have been reported: *N*^6^-methyladenine (6mA) and *N*^4^-methylcytosine (4mC). 6mA is the most prevalent form of methylation in prokaryotes, but its presence in mammalian cells is still in debate [92–96]. Similar to 6mA, 4mC is well known to exist in bacteria but its presence in eukaryotic genomic DNA remains unclear [95]. Beta-D-glucopyranosyloxymethyluracil (base J) is the first hypermodified base found in eukaryotic DNA. It was initially discovered in 1993 and has since been predominantly observed in kinetoplastids [97].

## RNA m^6^A modification sequencing technologies

m^6^A is the most prevalent internal modification in mRNA and has been intensively studied since its first identification in 1974 [98]. m^6^A is involved in RNA splicing, translation, stability, translocation, and high-level structure regulation [99, 100], and it has been linked to diverse developmental processes and cancers [18, 100–103]. m^6^A is enriched around stop codons and 3’ UTRs (3’ untranslated regions) in mammals. The installation of the modification is catalyzed by m^6^A methyltransferase complex proteins (so called “writers”), such as METTL3/14/16, RBM15/15B, ZC3H13, VIRMA, CBLL1, WTAP, and KIAA1429; the m^6^A recognition is accomplished by m^6^A-binding proteins (“readers”), such as YTHDF1/2/3, YTHDC1/2, IGF2BP1/2/3 and HNRNPA2B1 [17, 100, 104–109]. The demethylases (“erasers”), including FTO [110] and ALKBH5 [111], can remove m^6^A modifications.

Traditionally, the most widely used method for mapping m^6^A modifications is m^6^A-Seq, also called MeRIP-Seq, which is similar to MeDIP-Seq, using antibody enrichment [112, 113]. Other strategies based on the m^6^A antibody have also been developed, such as PA-Seq, miCLIP, m^6^A-Seq2, m^6^A-LAIC-Seq [114–117] (Fig. 2). As these antibody-based enrichment methods are largely influenced by the quality and intrinsic bias of the antibody, antibody-free m^6^A sequencing methods have subsequently been developed. MAZTER-Seq [118] and m^6^A-REF-Seq [119] (Fig. 2) use a methylation sensitive *E. coli* toxin and RNA endoribonuclease (MazF) which can recognize and cut the ACA motif sequence from 5’ sites. Although these methods can provide single-base resolution mapping of m^6^A sites, their preference for ACA sites means that only around 16–25% of all m^6^A sites across the whole transcriptome can be mapped. DART-Seq (deamination adjacent to RNA modification targets) [120] (Fig. 2) depends on cytidine deaminase APOBEC1 and m^6^A-binding YTH domain fusion protein to induce C-to-U deamination at sites adjacent to m^6^A modifications. However, this method requires cellular transfection which limits its application to primary cells and tissue samples. m^6^A-SEAL-Seq (FTO-assisted m6A selective chemical labeling method) [121] (Fig. 2) uses FTO to oxidize m^6^A into *N*^6^-hydroxymethyladenosin (hm^6^A), followed by dithiothreitol (DTT)-mediated thiol-addition reaction to generate *N*^6^-dithiolsitolmethyladenosine (dm^6^A): dm^6^A can then be labeled with biotin for pull-down and sequencing. m^6^A-label-Seq [122] (Fig. 2) is a metabolic labeling method that feeds the cells with a methionine analog, *Se*-allyl-L-selenohomocysteine, which can replace m^6^A with *N*^6^-allyladenosine (a^6^A). The a^6^A positions can be detected using iodination-induced misincorporation during reverse transcription. With this method, the authors demonstrated the detection of 2 479 and 3 808 m^6^A modification sites in HeLa and HEK293T cells, respectively. However, like DART-seq, m^6^A-label-Seq can only be applied to in vivo samples.

**Fig. 4:**
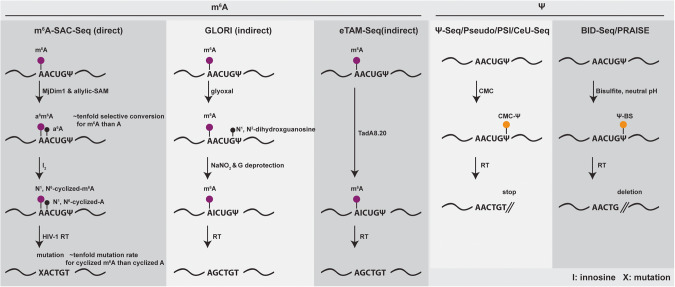
RNA m^6^A and Ψ modification detection sequencing technologies. Three methodologies for achieving single-base resolution and stoichiometric m^6^A sequencing have been developed: m^6^A-SAC-Seq, Glyoxal and nitrite-mediated deamination of unmethylated adenosines (GLORI), Evolved TadA-assisted *N*^6^-methyladenosine sequencing (eTAM-Seq). In the realm of Ψ sequencing, various approaches are based on CMC-Ψ, such as Ψ-Seq, Pseudo-Seq, pseudouridine site identification sequencing (PSI-Seq), and N_3_-CMC-enriched psedouridine sequencing (CeU-Seq). Notably, the most recent advancements in this field have yielded two innovative Ψ-BS based approaches: bisulfite-induced deletion sequencing (BID-Seq) and PRAISE.

Very recently, three single-base resolution and stoichiometric m^6^A sequencing methods have been developed. m^6^A-SAC-Seq [7] (Fig. 2) is the first enzyme dependent, direct, and quantitative m^6^A sequencing method. It uses the dimethyltransferase MjDim1 (*Methanocaldococcus jannaschii homolog*) which can convert m^6^A into a^6^m^6^A (*N*^6^-allyl, *N*^6^-methyladnosine) in the presence of allylic-S-adenosyl-methionine (SAM). With the iodination-induced misincorporation used in the m^6^A-label-Seq, a^6^m^6^A can induce mutations during reverse transcription, thereby detecting m^6^A at base-resolution (Fig. 4). However, MjDim1 exhibits strong sequence biases and poor selectivity of m^6^A over A, as well as high false positives. To achieve stoichiometric m^6^A measurement, calibration curves must be generated using spike-in probes with varying modification fractions. Nevertheless, m^6^A-SAC-Seq captures more than 10 000 m^6^A sites in HEK293, HeLa and HepG2 cells. This study revealed dynamic m^6^A modification stoichiometry during human hematopoietic stem and progenitor cells (HSPCs) differentiation.

Glyoxal and nitrite-mediated deamination of unmethylated adenosines (GLORI) [8] (Fig. 2) is the first indirect (similar to bisulfite sequencing for DNA methylation), base-resolution, and quantitative m^6^A sequencing method. This method is based on nitrous acid mediated adenosine deamination, which had been discovered half a century ago [123, 124]. To overcome nitrous acid’s low reaction efficiency towards A but a high rate of G deamination, GLORI uses glyoxal to protect G by forming an *N*^*1*^*, N*^*2*^-dihydroxyguanosine adduct (less than 3% G will convert after protection), which surprisingly also acts as a catalyst to boost the A to I (inosine) conversion (98–99%). m^6^A does not undergo nitrous-acid-mediated deamination, which allows it to be identified as the remaining A sites during sequencing (Fig. 4). GLORI captures 176 642 m^6^A modification sites in HEK293T cells (at 140x coverage) with around 40% median methylation level [8]. Most m^6^A sites reside in the canonical DRAC (D = G/A/T, R = A/G) motifs, while one third of m^6^A sites occur within clusters. As a chemical method, whether GLORI can be applied to low-input samples remains to be tested. Nevertheless, GLORI successfully charted the impact of hypoxia and heat shock conditions on the dynamic modification of m^6^A, thereby suggesting distinct regulatory mechanisms governing m^6^A’s role in gene expression through its influence on translation efficiency.

Evolved TadA-assisted *N*^6^-methyladenosine sequencing (eTAM-Seq) [9] (Fig. 2) is an enzymatic equivalent of GLORI, which uses a hyperactive transfer RNA adenosine deaminase (TadA) variant TadA8.20 to achieve up to 99% global adenosine-to-inosine deamination (Fig. 4). It identified 80 941 and 65 835 m6A sites in HeLa and mESC, respectively, and can be applied to RNA input quantities as low as 250 pg (10 cells) for loci-specific detection. It also unveiled an inverse relationship existing between m^6^A modification and the stability of mRNA molecules. However, due to the sensitivity of TadA8.20 to secondary structures, eTAM-seq requires control transcriptomes to eliminate false positives and may be less accurate at lowly methylated sites (<25%).

## RNA Ψ modification sequencing technologies

Ψ, also known as the ‘fifth nucleotide’ in RNA, was first identified in 1951. It is the most abundant modification in total RNA [125] and has been known to exist in tRNA, rRNA, and snRNAs for decades, whilst being recently found in mRNA. Ψ is generated by pseudouridine synthases (PUS) and accounts for ∼0.2% of uridine in mammalian mRNA [10, 126, 127]. Ψ’s base pairing is similar to uridine, being a structural isomer. Most methods for Ψ mapping rely on the labeling reaction of Ψ by *N*-cyclohexyl-*N’*-(2-morpholinoethyl) carbodiimide methyl-*p*-toluenesulfonate (CMC). The CMC-Ψ adduct can generate a stop signature during reverse transcription. These technologies, including Ψ-Seq [128], Pseudo-Seq [129], pseudouridine site identification sequencing (PSI-Seq) [130], and N_3_-CMC-enriched psedouridine sequencing (CeU-Seq) [126], however, lack stoichiometric information of Ψ (Fig. 2).

Recently, bisulfite-induced deletion sequencing (BID-Seq) [10] and PRAISE [11] (Fig. 2) have been developed using a bisulfite-mediated reaction on Ψ [131, 132]. Using reaction conditions at neutral pH, they can efficiently convert Ψ to Ψ-bisulfite (Ψ-BS) adduct without C to U conversion. The Ψ-bisulfite can cause deletion during reverse transcription, with fully modified Ψ generating about 70–90% detection rate (Fig. 4). With the help of calibration curves from spike-in controls, the BID-Seq quantified over 500 abundant Ψ modification sites in human HeLa, HEK293T, and A549 cells, which are enriched in the coding sequence (CDS) and 3’-UTR. In this study, Ψ modification is shown to be positively correlated with mRNA stability, expression and promotes read-through of stop codons [10]. The PRAISE study, on the other hand, identified 2 209 confident Ψ sites within the HEK293T cell line [11].

## Other RNA methylation modifications (m^1^A/m^5^C/hm^5^C/m^3^C/m^7^G)

Numerous other modifications exist in RNA, usually in low abundance. As an isomer of m^6^A, the established m^1^A-ID-Seq [133], m^1^A-Seq [134], m^1^A-MAP [135], and m^1^A-Seq-TGIRT [136] (Fig. 2) can be used to detect m^1^A modifications. Similar to DNA methylation, m^5^C, and hm^5^C have also been found in mRNA. m^5^C modification can be detected by modified bisulfite sequencing [137], Aza-IP [138], and miCLIP-Seq [139] (Fig. 2). m^7^G is another RNA methylation which can be detected by m^7^G-MeRIP-Seq [140], m^7^G miCLIP-Seq [141], AlkAniline‐Seq [142], m^7^G-MAP-Seq [143], and m^7^G-quant-Seq [144] (Fig. 2). 3-Methylcytidine (m^3^C) can be detected by AlkAniline‐Seq [142] and HAC-Seq [145] (Fig. 2).

## Third generation sequencing for DNA and RNA modifications

PacBio Single-Molecule Real-Time (SMRT) sequencing by Pacific Biosciences [25, 146, 147] and Oxford Nanopore sequencing [148–150] are novel third-generation technologies, which enable long-read sequencing and can directly detect native DNA and RNA modifications [12]. SMRT sequencing uses polymerase kinetics to discriminate different types of DNA modifications whilst nanopore sequencing utilizes the ionic current changes that occur when different nucleotides transit the nanopore channel to discriminate various DNA and RNA modifications. While the Nanopore platform has demonstrated its ability to detect various nucleic acid modifications, such as 5mC, 5hmC in DNA and m^6^A, m^5^C in RNA [151–157], the SMRT platform exhibits promise in the identification of RNA modification (m^6^A) through the analysis of fluorescent signal alterations during reverse transcription. However, its reliability currently remains limited [158, 159]. While they hold great potential for epigenomic and epitranscriptomic sequencing, at present, significant challenges and limitations remain, including high error rate, high cost, and high-sample input requirement. Currently, combining chemical or enzymatic reactions of DNA and RNA modifications with third generation sequencing could provide a solution to enhance their detection abilities [26, 160–163].

## Concluding remarks

In this review, we have described the various sequencing technologies for histone, DNA, and RNA modifications. A modification is best determined by an absolute quantitative sequencing method at base resolution. DNA methylation was traditionally achieved by bisulfite sequencing. Recent advances in this area resulted in EM-Seq and TAPS overcoming the issues of bisulfite sequencing. Compared to the more mature DNA epigenetic sequencing, recent efforts have started shifting to RNA epitranscriptomic sequencing. Very recently, breakthroughs such as m^6^A-SAC-Seq, GLORI, BID-Seq, and eTAM-Seq started to deliver base-resolution and quantitative sequencing of RNA modifications. Their efficiencies and accuracies are still lacking compared to DNA methylation sequencing methods, highlighted by the fact that different methods for the same modification often produce very distinct profiles (e.g., m^6^A and Ψ maps of the same cell line obtained by different sequencing methods showed low overlaps) [8–11]. A non-modified negative control (such as in vitro transcribed RNA) could be beneficial to eliminate false positives [8, 9, 11, 164]. We expect rapid development to continue in the field.

Future development of chemical methods and computational analysis in third-generation sequencing technologies will continue to improve their epigenomic and epitranscriptomic sequencing ability. It remains to be seen whether they can achieve the same accuracy and cost-effectiveness as next-generation sequencing in sequencing DNA and RNA modifications. Another potential of third-generation sequencing is for the simultaneous detection of multiple epigenetic or epitranscriptomic modifications in a single molecule [132, 165]. Such information could help reveal the interplay between different modifications.

With the rapid development of temporal/spatial DNA and RNA sequencing in recent years, including techniques such as Slide-Seq [166, 167] and Live-Seq [168], researchers have successfully examined the DNA and RNA molecules in their natural locations and in a dynamic manner. These advanced detection technologies also offer valuable insights into their potential application in uncovering epigenetic modifications. While the ability to investigate biological phenomena in real time and within their spatial context is of utmost significance for comprehensive understanding in the field of biology, it is tempting to envision future large-scale live cell temporal/spatial epigenetic sequencing to further enable biological discoveries.
